# Humoral Effect of SARS-CoV-2 mRNA vaccination with booster dose in solid tumor patients with different anticancer treatments

**DOI:** 10.3389/fonc.2023.1089944

**Published:** 2023-02-22

**Authors:** Chiara Piubelli, Matteo Valerio, Matteo Verzè, Fabrizio Nicolis, Carlotta Mantoan, Sonia Zamboni, Francesca Perandin, Eleonora Rizzi, Stefano Tais, Monica Degani, Sara Caldrer, Federico Giovanni Gobbi, Zeno Bisoffi, Stefania Gori

**Affiliations:** ^1^ Department of Infectious, Tropical Diseases and Microbiology, IRCCS Sacro Cuore Don Calabria Hospital, Negrar di Valpolicella, Vr, Italy; ^2^ Oncology Department, IRCCS Sacro Cuore Don Calabria Hospital, Negrar di Valpolicella, Vr, Italy; ^3^ Medical Direction Unit, Medical Direction, IRCCS Sacro Cuore Don Calabria Hospital, Negrar di Valpolicella, Vr, Italy; ^4^ Nurse Direction Unit, Nurse Direction, IRCCS Sacro Cuore Don Calabria Hospital, Negrar di Valpolicella, Vr, Italy

**Keywords:** SARS-CoV-2, Comirnaty, IgG, neutralizing antibodies, solid tumors, anticancer treatment, metastasis

## Abstract

**Introduction:**

Cancer patients are at risk for serious complications in case of SARS-CoV-2 infection. In these patients SARS-CoV-2 vaccination is strongly recommended, with the preferential use of mRNA vaccines. The antibody response in cancer patients is variable, depending on the type of cancer and antitumoral treatment. In solid tumor patients an antibody response similar to healthy subjects has been confirmed after the second dose. Only few studies explored the duration of immunization after the two doses and the effect of the third dose.

**Methods:**

In our study we explored a cohort of 273 solid tumor patients at different stages and treated with different anticancer therapies.

**Results and Discussion:**

Our analysis demonstrated that the persistence of the neutralizing antibody and the humoral response after the booster dose of vaccine was not dependent on either the tumor type, the stage or type of anticancer treatment.

## 1 Introduction

In case of SARS-CoV-2 infection, cancer patients are at increased risk of developing COVID-19 symptoms, serious complications and the need for hospitalization, with a mortality rate of 15-17% (1, 2). Moreover, if infected, these patients must suspend active anti-cancer treatments (with the exception of hormonal treatment) until the end of the quarantine period. For these reasons it is important to vaccinate cancer patients. The Italian Ministry of Health Recommendations have indicated the adult cancer patients (3, 4) among the priority groups for vaccination, being at greater risk of developing severe or lethal forms of COVID-19, such as patients who have been treated with immunosuppressive/myelosuppressive drugs (chemotherapy, immunotherapy, target therapy) or discontinued for less than 6 months and patients with advanced stage of disease not in remission.

In these patients, preferential use of messenger RNA (mRNA) vaccines has been indicated (5–7), with administration of two doses (the second three weeks after the first). The antibody response to the anti-SARS-CoV-2 vaccination could be influenced primarily by the state of immunodeficiency or secondarily by the pharmacological treatment. Published data have shown a significantly lower antibody response after the first dose in cancer patients (32-37%) than in non-oncological subjects (66%); after the second dose the antibody response reached 97-100% (8). Another study showed that, in the 232 enrolled cancer patients, only 29% developed an antibody response after the first vaccine dose compared to 84% in the 261 controls (p<0.001); however, after the second dose, a seroconversion rate of 86% was reported in cancer patients (9).

Subsequent scientific evidence suggested a potential benefit from a third additional dose of mRNA vaccine in cancer patients. Indeed in cancer patients, the development of a SARS-CoV-2 infection led to a further increase in antibody response (10–13). Moreover in immunocompromised patients undergoing organ transplantation, a third dose of vaccine significantly increased anti-SARS-CoV-2 antibodies, without severe side effects (14). In a phase I study enrolling 20 patients with cancer after a third dose of mRNA vaccine, no severe side effects were observed, while a modest increase in neutralizing antibodies was reported (15). In other small studies a benefit from the third dose of mRNA vaccine was shown (16–18).

Therefore EMA approved in September 2021 an additional third dose of anti-SARS-CoV-2 mRNA vaccine for cancer patients to be administered at least 28 days after the second dose. In Italy, the Ministry of Health has indicated the administration of the third dose (additional dose) in oncological or onco-haematological patients treated with immunosuppressive or myelosuppressive drugs or less than 6 months after discontinuing treatment (19).

The aim of this study was to evaluate the degree and the duration of the immune response 6 months after administration of two doses of anti-SARS-CoV-2 mRNA vaccine (Comirnaty, BioNTech/Pfizer) and the degree of immune response 3 weeks after administration of the third dose in a cohort of patients with solid tumors, at different stages and treated with different anticancer therapies.

## 2 Materials and methods

### 2.1 Study design and ethical approval

This retrospective study was conducted analyzing two sets of banked serum samples, longitudinally collected. The first set was collected 6 months after the first dose (T1) in a cohort of 273 cancer patients, who completed a two-doses vaccination cycle with BNT162b2 (Comirnaty). The second set was collected from the same cohort (150 patients) 3 weeks after the third dose (T2) of BNT162b2 vaccine. Serum samples were stored at -80°C in Tropica Biobank of the of the IRCCS Sacro Cuore Don Calabria Hospital.

All patients signed an informed consent and the study was approved by the local Ethics Committee (Comitato Etico per la Sperimentazione Clinica delle Province di Verona e Rovigo) on 13^TH^ June 2022 (study protocol n. 37382/2022).

The humoral response was evaluated by testing IgG antibodies against the receptor binding domain (RBD) of the S protein of SARS-CoV-2 virus.

### 2.2 Patient population

Serum samples were collected during routine visits from patients with solid tumor at the Oncology Department of IRCCS Sacro Cuore Don Calabria Hospital, from September to November 2021. Inclusion criteria for enrolling patients in the study were: age ≥18 years, diagnosis of solid tumor [I, II, III, IV stage, according to the AJCC Cancer Staging Manual classification scale (20),], Comirnaty vaccination (at least two doses), active anti-cancer treatment during vaccination or treatment discontinued for less than 6 months, signing of informed consent for use of sample and data.

### 2.3 Antibody analysis

Immunogenicity analysis was conducted by testing the SARS-CoV-2 IgG II Quant assay (Abbott, Ireland), used for the quantitative measure of IgG antibodies, including the neutralizing antibodies, against the receptor-binding domain (RBD) of the S1 subunit of the SARS-CoV-2 spike protein (IgG-RBD-S), in human serum. The automated assay was performed according to the manufacturer’s procedure, using the ARCHITET i2000 System (Abbott). The results are reported as binding antibody Unit/mL (BAU/mL), according to the following interpretation: BAU/mL<7.1 = negative, BAU/mL≥7.1 = positive.

### 2.4 Statistical analysis

Statistical analyses were performed using R software version 4.1.0 (21).

Differences in immunologic responses were assessed using the Mann-Whitney U test or the Kruskal-Wallis test for unpaired data at a specific time point, whereas the Wilcoxon signed-rank test was applied to assess differences within groups for paired data at different time points. In addition, a multiple linear regression model was used to evaluate the association of the patient related factors (sex, age at vaccination, metastatic disease, treatment, tumor type) with the outcome, represented by the increment in IgG-RBD-S from T1 to T2 (Δ_IgG-RBD-S_).

A p-value < 0.05 was considered as statistically significant.

## 3 Results

### 3.1 Patient characteristics

A total of 505 samples from a cohort of 273 oncologic patients were analyzed, 273 samples were available 6 months after the primary series of COVID-19 vaccine (T1) of Comirnaty vaccine and 150 samples 3 weeks after the third dose (T2) of the same type of vaccine.

Patients’ characteristics are summarized in **Table 1**. Briefly, all the patients completed at least one two-dose vaccination cycle. The median age at diagnosis was 61.0 years (IQR 51.0-68.0) and 61.5% were female. At T1, metastatic disease was present in 112 (41%) of patients, 35 (13%) cases were stage I, 51 (19%) stage II, and 67 (24%) stage III.

**Table 1:** Demographic and clinical characteristics of patients at different time points.

CHARACTERISTIC	T1	T2
Number of patients	273 (100%)	150 (100%)
Sex		
*Male*	105 (38%)	63 (42%)
*Female*	168 (62%)	87 (58%)
Age at diagnosis (years)	61.0 [51.0, 68.0]	61.0 [51.0, 68.0]
Age at vaccination (years)	64.0 [55.0, 71.0]	64.0 [53.2, 72.5]
Metastatic disease		
*No*	161 (59%)	81 (54%)
*Yes*	112 (41%)	69 (46%)
Stage		
*I*	35 (13%)	16 (11%)
*II*	51 (19%)	30 (20%)
*III*	67 (25%)	30 (20%)
*IV*	113 (41%)	71 (47%)
*Missing*	7 (3%)	3 (2%)

Descriptive statistic report median [IQR] for continuous variables or n (%) for categorical variables.

**Figure 1:**
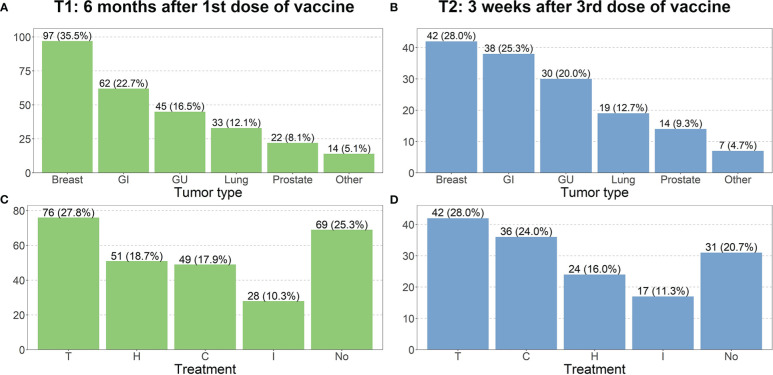
Distribution of tumor type and treatment at different time point. Charts **(A–D)** refer respectively to T1 and T2. (GI, Gastrointestinal; GU, Genitourinary; C, Chemotherapy; H, Hormone therapy; I, Immunotherapy; T, Target therapy; No, No treatment).

As reported in =**Figure 1A**, at T1 the most frequent tumor types were breast (35%), gastrointestinal (23%), genitourinary (16%), lung (12%) and prostate cancer (8%). In the group “other” (5.1%), the following cancer type were included: brain tumor, melanoma, head and neck tumor, sarcoma and apudoma. These percentages slightly changed at T2 (**Figure 1B**) due to a reduction of the population.

Most patients were receiving antitumoral therapy at the time of the first, second and third dose of vaccine administration. At T1, 28% of patients were receiving target therapy (+ or – hormone therapy), 19%, 18% and 10% were receiving hormone, chemo- (+ or – hormone therapy) or immunotherapy respectively (**Figure 1C**). At T2, 28% of patients were receiving target therapy (+ or – hormone therapy), 24% chemotherapy (+ or – hormone therapy), 16% hormone therapy and 11% immunotherapy (**Figure 1D**).

### 3.2 Vaccination adverse events

Data collected after the second dose reported only mild side effects caused by the vaccination, with pain at the injection site as the most frequent (36%) followed by fever with temperature ≥38°C (22%). No data was collected after the third dose, but sporadic cases of transient axillary lymphadenopathy were observed.

### 3.3 Antibody measurements after vaccination

At T1 the median IgG-RBD-S value in the analyzed population was 96.7 BAU/mL (IQR 36.6 – 215.8) and only the 5% of patients showed a negative value (BAU/ml < 7.1). After the administration of the third dose at T2, the median IgG-RBD-S value markedly increased to 2988.5 BAU/mL (IQR 1073.2 – 6232.2) and only 1% (1 patient) had a negative value.

Different parameters were evaluated by statistical analysis, in order to understand if the antibody values or increment after the booster dose were influenced by the type of tumor, the stage, the presence of metastatic disease or antitumoral treatment.

**Figure 2:**
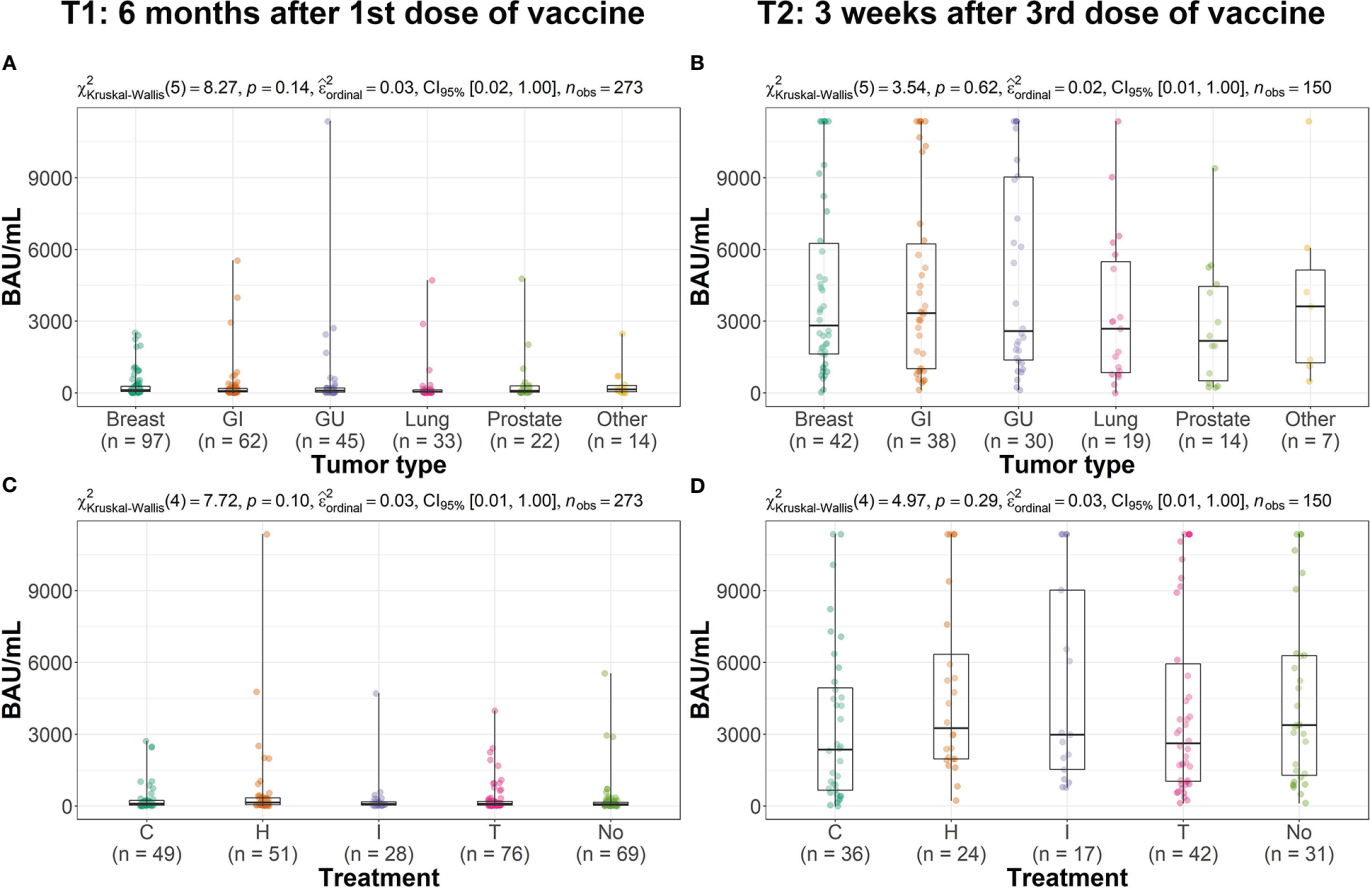
Distribution of the IgG-RBD-S value, expressed in BAU/mL, across groups. Box plots **(A–D)** refer respectively to T1 and T2. Each box covers the interquartile range; the splitting line is the median and whiskers represent minimum and maximum values. Statistical significance, set at p-value <0.05, was assessed using the Kruskal-Wallis test. (GI, Gastrointestinal; GU, Genitourinary; C, Chemotherapy; H, Hormone therapy; I, Immunotherapy; T, Target therapy; No, No treatment).

Comparison analysis among different tumor types (breast, gastrointestinal, genitourinary, lung, prostate cancer and other) showed no significant difference in the IgG-RBD-S antibodies level either at T1 (**Figure 2A**) or T2 (**Figure 2B**).

Similarly, no differences were found among different groups of antitumoral treatment (target therapy, hormon, chemo- or immunotherapy, **Figures 2C, D**).

**Figure 3:**
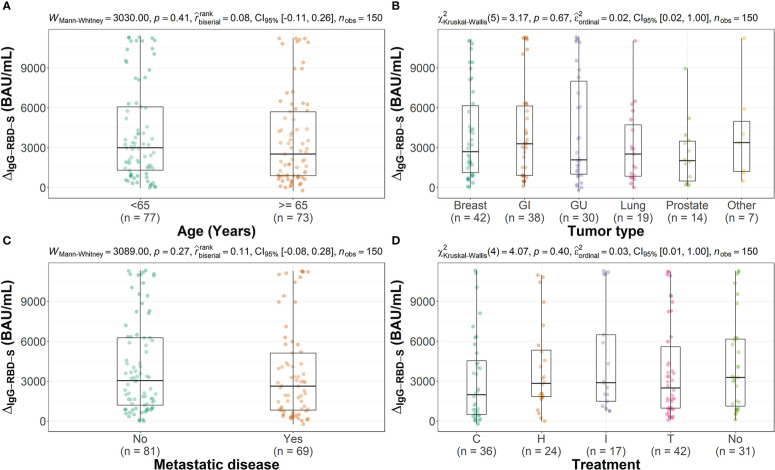
Distribution of the increment in IgG-RBD-S value (IgG-RBD-S_T2_ - IgG-RBD-S_T1_), expressed in BAU/mL, across groups for different characteristics. (**A**: Age, **B**: Tumor type, **C**: presence of metastatic disease, **D**: type of treatment) Each box covers the interquartile range; the splitting line is the median and whiskers represent minimum and maximum values. Statistical significance, set at p-value <0.05, was assessed using the Mann-Whitney U test or the Kruskal-Wallis test. (G, Gastrointestinal; GU, Genitourinary; C, Chemotherapy; H, Hormone therapy; I, Immunotherapy; T, Target therapy; No, No treatment).

For the 150 subjects for which both T1 and T2 samples were available, the increase in IgG-RBD-S antibodies value after the third dose was evaluated for paired data, considering the different type of tumor or treatment, the presence of a metastatic disease and age. For all the analyzed conditions a statistically significant increase in the IgG-RBD-S antibodies was observed after the third dose of vaccine, with an overall fold change of 30.5 (**Table 2**). Moreover, as reported in **Figure 3**, no influence of age, tumor type, metastatic disease or treatment type on the humoral response after the third dose was observed. No sex related differences were observed too (Mann-Whitney test, p=0.61, data not shown).Furthermore these data were confirmed by a multiple linear regression analysis, used to evaluate the association of the patient related factors (sex, age at vaccination, metastatic disease, treatment, tumor type) with the outcome, represented by the increment in IgG-RBD-S from T1 to T2 (data reported in **Supplementary Table S1**). The multiple regression analysis, with all the factors included in the model, did not reach significant association between the factors and the outcome.

**Table 2:** IgG-RBD-S value across groups for paired data at T1 (6 months after primary series of COVID-19 vaccine of vaccine) and T2 (3 weeks after third dose of vaccine).

CHARACTERISTIC		N (%)	gG-RBD-S(T1)^a^ (BAU/mL)	IgG-RBD-S(T2)^a^ (BAU/mL)	p-value^b^
**Overall**		150 (100%)	98.1 [38.4 - 210.7]	2988.5 [1073.2 - 6232.2]	<0.001
**Sex**	Male	63 (42.0%)	76.2 [24.7 - 168.2]	2730.9 [955.4 - 5558.6]	<0.001
	Female	87 (58.0%)	122.7 [46.7 - 273.3]	3054.5 [1408.1 - 6367.7]	<0.001
**Age at vaccination**	< 65 years	77 (51.3%)	120.3 [38.8 - 218.1]	3068.2 [1525.1 - 6358.9]	<0.001
	≥ 65 years	73 (48.7%)	84.3 [36.6 - 206.6]	2679.5 [927.9 - 5919.0]	<0.001
**Metastatic disease**	No	81 (54.0%)	121.0 [39.5 - 220.4]	3374.6 [1394.4 - 6557.4]	<0.001
	Yes	69 (46.0%)	93.6 [32.3 - 204.6]	2693.8 [907.7 - 5346.9]	<0.001
**Stage**	I	16 (10.7%)	115.7 [20.7 - 238.4]	1763.9 [1028.1 - 4102.1]	<0.001
	II	30 (20.0%)	82.3 [46.4 - 209.6]	3223.4 [1888.6 - 6486.4]	<0.001
	III	30 (20.0%)	130.4 [39.6 - 266.8]	4111.1 [1379.7 - 9144.9]	<0.001
	IV	71 (47.3%)	93.6 [33.7 - 196.7]	2693.8 [911.8 - 5291.6]	<0.001
	Missing	3 (2.0%)	–	–	–
**Treatment**	Chemotherapy	36 (24.0%)	69.0 [30.7 - 179.5]	2356.6 [656.0 - 4940.9]	<0.001
	Hormone therapy	24 (16.0%)	215.3 [80.1 - 405.4]	3247.9 [1970.0 - 6337.1]	<0.001
	Immunotherapy	17 (11.3%)	76.9 [38.5 - 157.8]	2977.6 [1525.1 - 9022.5]	<0.001
	Target therapy	42 (28.0%)	120.1 [56.4 - 208.3]	2615.4 [1031.4 - 5941.7]	<0.001
	No treatment	31 (20.7%)	74.4 [23.7 - 175.9]	3374.6 [1280.3 - 6281.4]	<0.001
**Tumor type**	Breast	42 (28.0%)	176.1 [64.4 - 339.0]	2823.2 [1631.1 - 6248.9]	<0.001
	Gastrointestinal	38 (25.3%)	74.1 [30.6 - 134.3]	3337.3 [1014.3 - 6224.9]	<0.001
	Genitourinary	30 (20.0%)	124.7 [44.7 - 210.2]	2588.9 [1379.7 - 9024.4]	<0.001
	Lung	19 (12.7%)	53.3 [29.5 - 96.9]	2679.5 [851.1 - 5487.3]	<0.001
	Prostate	14 (9.3%)	166.3 [28.9 - 291.7]	2179.2 [508.1 - 4456.0]	<0.001
	Other	7 (4.7%)	133.9 [50.6 - 173.5]	3620.6 [1257.2 - 5139.1]	<0.001

a Median [Interquartile range].

b Wilcoxon signed-rank test.

## 4 Discussion

In the present study, we collected serum samples from a cohort of solid cancer patients with any stage of disease during treatment with immunosuppressive/myelosuppressive drugs (chemotherapy, immunotherapy, target therapy) or discontinued for less than 6 months and patients with advanced stage of disease not in remission. We evaluated the persistence of the humoral response to the BNT162b2 mRNA vaccine 6 months after the completion of the two-dose administration and the boosted answer produced by the third dose. In our population, solid tumors were mainly represented by breast, gastrointestinal, genitourinary, lung and prostate cancer. The most representative treatment was target therapy, followed by chemo- or hormon and immuno-therapy. For a group of patients instead (20%) the therapy was stopped by less than 6 months at the moment of the third dose vaccination.

At T1, 95% of patients still showed positive level of IgG-RBD-S antibodies, with a median value similar to that reported in literature for healthy subjects (22) and in line with that reported by other studies on cancer patients’ populations (23, 24). The 5% of patients showing negative IgG-RBD-S antibodies were spread across all tumor types and treatment groups. Our analysis demonstrated that the persistence of the neutralizing antibodies was not dependent on either the tumor type, or type of anticancer treatment (**Figures 2A, C**).

After the third vaccine dose, a significant (on average 30.5-fold) increase in antibody levels was observed in the entire population analyzed (**Table 2**), indicating the presence of an enhanced active response, as observed in other studies with smaller cohorts of solid tumors patients (15). At T2 the antibody level across tumor and treatments groups are similar (**Figures 2B, D**) and we didn’t find any significant difference neither according to tumor nor treatment type. The only subject who did not elicited IgG-RBD-S antibodies after the third dose of the Comirnaty vaccine was affected by squamous lung cancer associated with an untreated chronic lymphocytic leukemia (CLL). CLL patients are characterized by immunosuppression due to disease itself and cytotoxic treatments, so the absence of an immunological response was most probably due to CLL than to the solid lung tumor (25–27). Literature data indicated that this category of immunocompromised patients produce an antibody response after a standard vaccination cycle only in about 50% of cases, depending also on the treatment (26, 28, 29), and the response to the third dose is even low in non-responder patients (15%), particularly in who had received anti-CD20 immunotherapy within a year from vaccination (3.6%) (28, 30). One study pointed out a better humoral answer in CLL patients vaccinated with Moderna than other vaccines (26), but considering that anyway the response is not always successful for all CLL patients, a strict preventive strategy should be applied for these patients.

For all tumor types, stages and treatments the increase in antibody levels after the third dose was statistically significant and this was true for both men and women and in both age groups (<65 and >=65). We observed that neither the tumor nor the type of treatment influenced the magnitude of the humoral response after the booster dose (**Figures 3B, D**) and that the response was not influenced by age or by the presence of metastases (**Figures 3A, C**).

This study has some limitations, such as the retrospective design, the lack of a follow-up point of antibody measurement after T2 and the absence of information about SARS-CoV-2 infections before vaccination primary cycle, as well as about some possible confounding factors, such as comorbidities. Moreover, the analysis was limited to the humoral response, since no lymphocytes samples were collected for immune memory evaluation. The evaluation of T-cell response should be monitored in future vaccination cycles, since some papers report a diminishing effect on T-cell specific activation after the third dose of SARS-CoV-2 mRNA vaccines respect to the previous two doses in cancer patients, suggesting a possible T cell exhaustion, due to repetitive priming to the same antigen (15, 31).

To conclude, our data strongly confirms that SARS-CoV-2 Comirnaty vaccination is able to elicit a significant humoral response in patients with solid tumors, irrespective of the tumor type and therapy.

## Data availability statement

The datasets presented in this study can be found in online repositories. The names of the repository/repositories and accession number(s) can be found below: https://zenodo.org/badge/DOI/10.5281/zenodo.7289234.svg.

## Ethics statement

The studies involving human participants were reviewed and approved by Comitato Etico per la Sperimentazione Clinica delle Province di Verona e Rovigo (study protocol n. 37382/2022). The patients/participants provided their written informed consent to participate in this study.

## Author contributions

SG, CP, FP, FG, conceived and designed the study. SG performed clinical evaluation and patient recruitment. MVe, FN, CM, SZ, organized patients vaccination and blood collection. ER, ST, MD performed sample collection and analysis. MVa performed data curation and statistical analysis. CP, SG and MVa wrote the manuscript. ZB, FG, and FP critically reviewed the data and manuscript. All authors read, critically revised, and approved the manuscript.
