# Supplementary material for: Humoral Effect of SARS-CoV-2 mRNA vaccination with booster dose in solid tumor patients with different anticancer treatments
Source: Front Oncol. 2023 Feb 22;13:1089944. doi: 10.3389/fonc.2023.1089944 (PMC9992722; doi:10.3389/fonc.2023.1089944)
Supplement: Supplementary file 1 [file Table_1.docx]

**Table S1:** results of a multiple linear regression model, used to evaluate the association of the patient related factors (sex, age at vaccination, metastatic disease, treatment, tumor type) with the outcome, represented by the increment in IgG-RBD-S from T1 to T2 (∆_IgG-RBD-S_).

| CHARACTERISTIC | | Coefficient | 95% CI^1^ | p-value |
| --- | --- | --- | --- | --- |
| Sex | F | - | - |  |
|  | M | -88 | -1794, 1617 | >0.9 |
| Age at vaccination | < 65 years | - | - |  |
|  | ≥ 65 years | -240 | -1487, 1007 | 0.7 |
| Metastatic Disease | No | - | - |  |
|  | Yes | 14 | -1284, 1312 | >0.9 |
| Treatment | Chemotherapy | - | - |  |
|  | Hormone therapy | 1259 | -777, 3295 | 0.2 |
|  | Immunotherapy | 1689 | -550, 3928 | 0.14 |
|  | Target therapy | 394 | -1271, 2059 | 0.6 |
|  | No treatment | 619 | -1224, 2463 | 0.5 |
| Tumor type | Breast | - | - |  |
|  | Gastrointestinal | 499 | -1661, 2659 | 0.6 |
|  | Genitourinary | 356 | -1471, 2184 | 0.7 |
|  | Lung | -974 | -3328, 1379 | 0.4 |
|  | Prostate | -1421 | -4417, 1576 | 0.4 |
|  | Other | -37 | -3364, 3291 | >0.9 |
| ^1^CI = Confidence Interval | | | | |
